# A database of words and pictures for investigating phonological-semantic processing and interaction in language processing: Validation and application

**DOI:** 10.1371/journal.pone.0336981

**Published:** 2025-11-17

**Authors:** Laura Anna Ciaccio, Luigi Grisoni, Sabrina Nieswand, Friedemann Pulvermüller

**Affiliations:** 1 Brain Language Laboratory, Department of Philosophy and Humanities, Freie Universität Berlin, Germany; 2 Department of Brain and Behavioral Sciences, Università di Pavia, Italy; 3 Center for Translational Neurophysiology of Speech and Communication, Istituto Italiano di Tecnologia, Ferrara, Italy; 4 Cluster of Excellence “Matters of Activity. Image Space Material”, Humboldt Universität zu Berlin, Germany; 5 Berlin School of Mind and Brain, Humboldt Universität zu Berlin, Berlin, Germany; 6 Einstein Center for Neurosciences, Berlin, Germany; Education University of Hong Kong, HONG KONG

## Abstract

An increasing number of studies aim to directly compare aspects of linguistic processing in word production and comprehension using controlled experiments with shared stimuli across modalities. However, the available resources allowing for such investigations are scarce, particularly for languages other than English. To address this gap, we present an open-source database of 151 German words and related picture stimuli. The items are drawn from two well-investigated semantic categories – animals and tools – and differ phonologically by either starting with a labial or a coronal speech sound. The database includes human ratings for depictive accuracy, familiarity, action relatedness, valence, arousal of the pictures, and familiarity, action relatedness, valence, arousal, and imageability of the words. In a set of analyses, we show that the ratings we collected have good reliability and external validity, and align well with previously obtained data from lexical and pictorial databases. The database is complemented with comprehensive information about phonetic/phonological, lexical, morphological, and semantic properties of the words, as well as about luminosity and visual complexity of the corresponding pictures. We finally present a matched stimulus set obtained from the database, as a ready-to-use resource for psycho- and neurolinguistic studies examining semantic and phonological processing along with their interaction in language production and comprehension. The word-picture database, together with the matched stimulus set, are freely available at https://osf.io/k64nh/.

## Introduction

In language production research, a growing number of studies seek to directly compare word production and comprehension in the context of controlled experiments involving the same items for both modalities [1–3]. To compare production and comprehension, these studies often use experimental manipulations *within* each modality [2,3], allowing the researchers to obtain behavioral or neurobiological correlates unrelated to physical stimulation due to the different input types. These manipulations may focus on specific cognitive-linguistic processes such as phonological or semantic access and memory; ideally, more than one of these processes are tested, varying them orthogonally. This allows to address the important psycholinguistic topic of the time course of first access to different types of language related information, so as to draw possible inferences on the seriality/cascading or parallelism of such information access [4].

A well-established method to investigate language production is the picture naming task [see, e.g., 5–7], as this is supposed to mimic best the cognitive processes involved in natural language production (i.e., from conceptual processing, lexical access, to articulation) [8–10]. In order to design a picture naming experiment, it is necessary to draw items from sets of pictures that (i) can be easily recognized, (ii) are named with a high level of agreement between individuals, and (iii) contain an adequate number of items from each of the linguistic subcategories of interest, to allow comparisons between subsets (e.g., at semantic level, between two semantic categories). Importantly, these subsets of items also need to be matched on various relevant properties; this involves not only the word items, but, crucially, also several properties of the corresponding pictures to be named.

In this paper, we present an open access database containing pictures and corresponding words, which we developed for research on production and comprehension in *German.* The database complements existing resources for German [picture databases: 11–14; words’ valence and/or arousal ratings: 15–17] by targeting specific semantic or phonological categories, therefore allowing for factorial designs focusing on clear binary distinctions at the semantic or phonological level, or, ideally, orthogonalizing both factors. It was conceived to suit the needs of neurophysiological and imaging studies, for which clear binary contrasts are especially relevant, but is, of course, well suited for behavioral studies too.

The database contains items belonging to two different semantic categories, i.e., animal and tool words. These semantic categories were chosen because several studies, employing different techniques, showed that they elicit distinguishable, category-specific brain activations [see, e.g., 3, 18–24]. Items additionally vary phonologically in terms of place of articulation (PoA), so that they either start with tongue-related (coronal) or lip-related (bilabial or labio-dental) sounds, a distinction for which recent studies have shown dissociations both in language production [4,25] and comprehension [26–29; for a review, see 30]. The database lends itself to additional phonetic/phonological contrasts, namely manner of articulation (MoA, especially fricatives and plosives), and voicing, which have been investigated in several studies, focusing both on language perception and production [25,31–33].

The database contains information about a series of phonetic/phonological, lexical, and morphological properties of the words, as well as visual complexity and luminosity measures of the pictures. For both words and pictures, we then collected ratings on several additional relevant semantic or conceptual dimensions: action-relatedness, arousal, valence, and imageability for the words; depictive accuracy, familiarity, action-relatedness, arousal, and valence for the pictures. To the best of our knowledge, this is the first database with action-relatedness norms of words or pictures in German. Furthermore, of all the available databases (on any language), this is the only one comprising ratings for action-relatedness, arousal, and valence for *both* words and their corresponding pictures, collected with the same method and within the same study.

The database was employed to design a study by Grisoni and colleagues investigating the earliest brain indicators of phonological, semantic, and pragmatic access and their possible interaction [34]. Therefore, along with presenting the general properties of the database as well as its validation, we additionally provide an overview of the specific stimulus set used in that study. This should illustrate an application of the database for psycholinguistics/neurolinguistics research, and possibly provide researchers with a ready-to-use stimulus set for further investigations of phonological and semantic processing and their interaction in the receptive and production domains.

## Materials

### General overview

The database contains 151 words and the corresponding 151 pictures. Henceforth, the term ‘item’ is used to refer to the concept associated to each word-picture pair, whereas the terms ‘word’ and ‘picture’ are used to distinguish between these two classes of stimuli. Items belong to two semantic categories: 69 are animal nouns and 82 are tool nouns. Animals could be further categorized as 38 mammals, 13 birds, 9 insects, 2 aquatic invertebrates, 2 fish, 2 reptiles, and 3 belonging to other subcategories. Tools could be further categorized as 16 kitchen utensils/appliances, 6 weapons, 5 cutting tools, 5 electronic devices, 5 office tools, 4 construction tools, 4 containers, 4 art/craft tools, 3 measuring tools, 3 sports objects, 3 writing instruments, 2 household items, 2 cleaning tools, 2 musical instruments, 2 optical instruments, and 16 items in additional miscellaneous categories. As for the phonological level, 77 items start with a ‘labial’ and 74 with a ‘coronal’ sound (see details under ‘Phonetic/phonological properties’). All the properties contained in the database are described in the sections below, with detailed information on how these were retrieved, coded, or collected. Table 1 provides an overview of the columns of the database and a brief explanation of the information that each of them contains. The database is freely available at our project’s OSF directory: https://osf.io/k64nh/. All analyses were performed in R (version 4.4.1) [35].

**Table 1:** Structure of the word-picture database.

Column name	Explanation
word	The word item
word_noumlaut	The word item, without umlauts and special characters
word_downcased	The word item, all in lower case, including first letter (which in German nouns is otherwise in capital letters)
english_equivalent	The word’s English translation or equivalent
semantic_condition	Semantic category of the word (animal, tool)
n_letters	Number of letters
n_phonemes	Number of phonemes or phonological segments (dipthongs, long vowels, and affricates are counted as 1 segment)
n_syllables	Number of syllables
voicing	Voicing of the word’s first phoneme (voiced, voiceless)
PoA	Place of articulation of the word’s first phoneme (labial, coronal)
MoA	Manner of articulation of the word’s first phoneme
initial_cluster	Word’s initial consonant cluster is complex = 1; otherwise = 0
complex_onsets	Word contains one or more complex syllable onsets = 1; otherwise = 0
complex_codas	Word contains one or more complex syllable codas = 1; otherwise = 0
morphologically_complex	Word is morphologically complex = 1; otherwise = 0
lemma_frequency_zipf	Word’s lemma frequency
bigram_frequency_corpus_zipf	Word’s bigram frequency, across all corpus tokens
wordform_downcased_frequency_zipf	Word’s word form frequency (irrespective of case)
pic_naming_correct_abs	Number of correct answers in the picture naming task
pic_naming_correct_prop	Proportion of correct answers in the picture naming task
pic_familiarity_mean	Mean of familiarity ratings (picture)
pic_familiarity_sd	Standard deviation of familiarity ratings (picture)
pic_depictive_accuracy_mean	Mean of depictive accuracy ratings (picture)
pic_depictive_accuracy_sd	Standard deviation of depictive accuracy ratings (picture)
pic_action_related_mean	Mean of action relatedness ratings (picture)
pic_action_related_sd	Standard deviation of action relatedness ratings (picture)
pic_arousal_mean	Mean of arousal ratings (picture)
pic_arousal_sd	Standard deviation of arousal ratings (picture)
pic_valence_mean	Mean of valence ratings (picture)
pic_valence_sd	Standard deviation of valence ratings (picture)
word_action_related_mean	Mean of action relatedness ratings (word)
word_action_related_sd	Standard deviation of action relatedness ratings (word)
word_arousal_mean	Mean of arousal ratings (word)
word_arousal_sd	Standard deviation of arousal ratings (word)
word_valence_mean	Mean of valence ratings (word)
word_valence_sd	Standard deviation of valence ratings (word)
word_image_mean	Mean of imageability ratings (word)
word_image_sd	Standard deviation of imageability ratings (word)
pic_name	File name (picture)
filesize_zip_png	Size of zip-compressed file (picture) as an estimator of visual complexity
filesize_jpeg	Size of JPEG-compressed file (picture) as an estimator of visual complexity
mean_pixel_values	Mean pixel values (picture) as an estimator of picture luminosity

*Words*. Words are, on average, 2.07 syllables long (SD 0.65, range 1–4), with mean number of letters and phonemes being, respectively, 6.54 (SD 1.96, range 3–12) and 5.61 (SD 1.71, range 2–11). Most words are morphologically simple; 41 items contain more than one morpheme (33 compound words and 8 *-er* derivations). We extracted lemma frequency, (case-insensitive) word-form frequency, and bigram frequency (i.e., the sum of the frequency of each bigram of a word) from the dlex database [dlexDB, 36], which is a German annotated lexical database derived from a corpus of about 120 million tokens. Four of our word items are not listed in dlexDB. For these items, lemma and word-form frequency were coded as 0, and bigram frequency as ‘NA’. Frequencies are expressed in the zipf-scale [37], which is a log-10 scale spanning approximately from 1 to 7 (1 = 0.01 per million frequency; 7 = 10,000 per million frequency). Mean lemma, word-form, and bigram frequencies are, respectively, 3.21 (SD 0.91, range 0–5.10), 3.00 (SD 0.90, range 0–5.10), and 8.36 (SD 0.27, range 7.39–8.86). Figures A1 and A2 in the Appendix illustrate the distributions of these lexical properties.

*Pictures*. All pictures are drawn by a local artist commissioned by the Brain Language Laboratory of the Freie Universität of Berlin. The artist was instructed to use the same style for all the pictures, and to use a style as similar as possible to that used in another well-known database [38]. All pictures are black-lined drawings on a transparent background, with a size of 2362 × 2362 pixels (DPI = 28.35 pixels/cm), in PNG format. For two words (‘Wischmopp’ and ‘Pfeil’), the artist created two different pictures, which were both included in the first rating study. Therefore, the ratings were performed on a total of 153 pictures and 151 words. For the presentation of the database in the current paper, and the corresponding analyses we report here, we kept the picture (out of the two pictures presented) that obtained the highest naming consistency score. The two excluded pictures can be found in the database. All pictures are available at our project’s OSF repository: https://osf.io/k64nh/.

### Phonetic/phonological properties

As illustrated in Table 1, the database contains dedicated columns for several phonetic/phonological properties. Words start with one of 14 different consonants (/b/,/d/,/f/,/l/,/m/,/n/,/p/,/pf/,/s/,/∫/,/t/,/ts/,/v/,/z/). Depending on the PoA of the initial consonant, i.e., whether the articulation of the initial consonant mainly involves the lips or the tip of the tongue, items are coded as, respectively, ‘labial’ (N = 77) or ‘coronal’ (N = 74). More specifically, the ‘labial’ group includes bilabial and labio-dental sounds, whereas the ‘coronal’ category includes alveolar and post-alveolar sounds. Our items cover voiced (N = 70) and voiceless (N = 81) sounds, from five different MoAs: fricative (N = 56), plosive (N = 53), lateral (N = 12), nasal (N = 20), and affricate (N = 10). Note that the number of voiced and voiceless sounds is comparable across sub-groups of words, both if we divide them by PoA (coronal voiced: 33, coronal voiceless: 41, labial voiced: 37, labial voiceless: 40), and if we divide them based on the MoA, i.e., on the fricative/plosive distinction (fricative voiced: 18, fricative voiceless: 38, plosive voiced: 20, plosive voiceless: 33).

In addition to a classification of words according to phonological properties of their initial consonants, the words are annotated for the presence of complex consonant clusters. Thirty-six of the words start with a complex consonant cluster, i.e., with more than one consonant sound in a row. Forty-seven items contain a complex syllable onset, while 30 contain a complex syllable coda (irrespective of syllable position within the word).

### Visual complexity and luminosity (pictures)

For all pictures, we computed two visual complexity measures and one luminosity measure. Unlike other existing databases [11], which used subjective ratings for visual complexity, we opted for objective measures. Objective measures of visual complexity have the advantage of not being confounded with concept familiarity, word frequency, or age of acquisition, while at the same time being highly correlated with subjective ratings [39,40]. These measures have been shown to affect naming accuracy [40] and, at least in some languages, naming latencies [41]. Following previous studies, our two measures of visual complexity were the file size in KB of, respectively, the JPEG and the zip-compressed files obtained from the original pictures in PNG format [39,40,42,43]. We operationalized luminosity as the mean of the RGB pixel values of the original image [see [43], for a similar procedure]. This, in essence, provides an indication of how much blackness the image contains. Because white corresponds to the RGB values [255, 255, 255] and black to [0, 0, 0], a high mean pixel value corresponds to high luminosity.

Pictures have a mean zip-compressed file size of 412.79 KB (SD 522.97, range 65–3,672) and a mean JPEG-compressed file size of 443.03 KB (SD 287.67, range 142-1,980). For both measures, most items have a file size below 1,000 KB, and those exceeding this threshold are rather outliers (9 items for zip-compressed and 6 items for JPEG-compressed file size). The two measures of visual complexity are, as expected, very strongly positively correlated with each other (r = 0.947, 95%CI [0.928, 0.961], *p* < .00001). As for luminosity, the means of the pictures’ mean of RGB pixel values is 247.98 (SD 6.99, range 204.15–253.44). Figure A3 in the Appendix illustrates the distributions of these measures.

**Fig 1:**
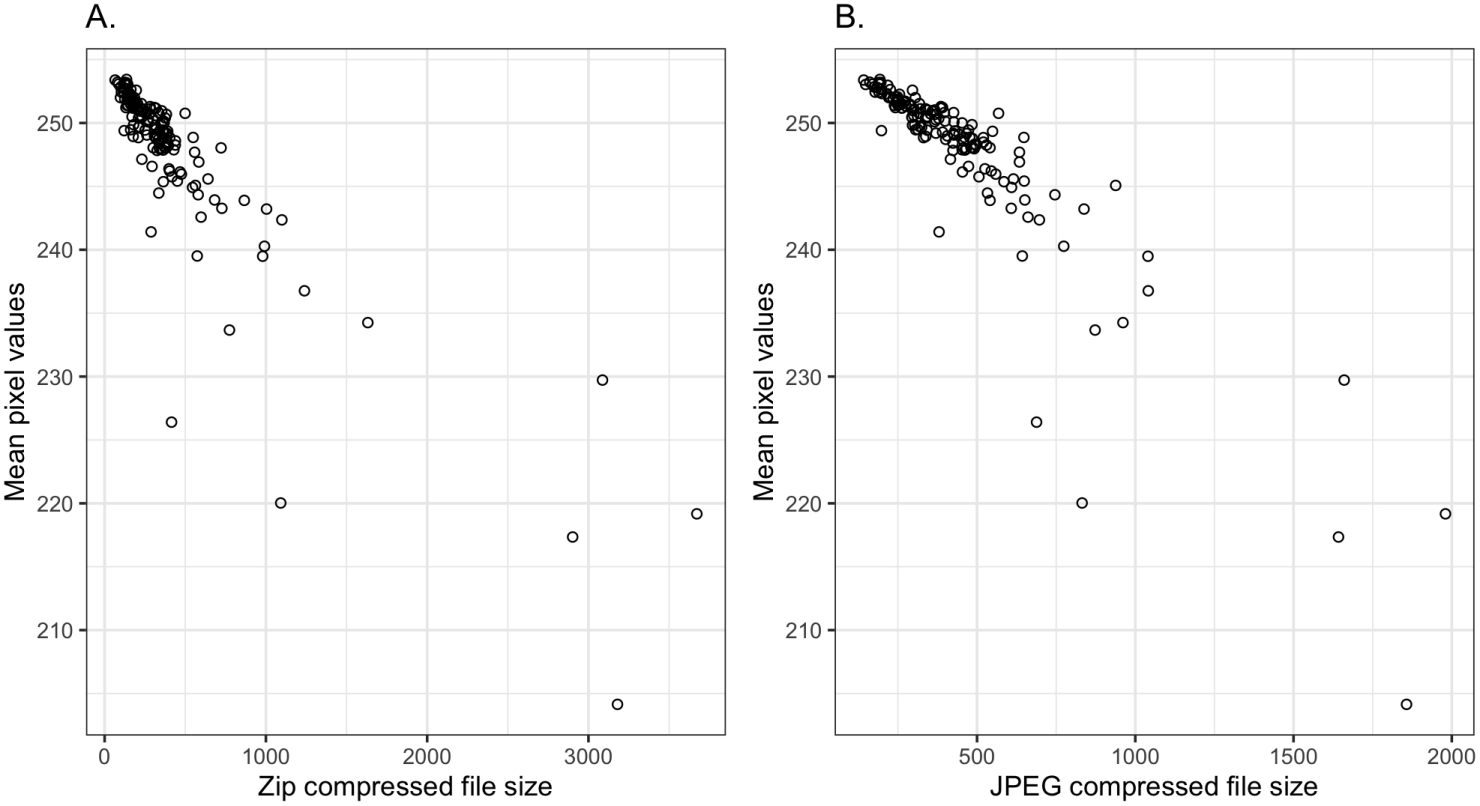
Correlations between picture complexity and luminosity measures. A. Correlation between zip-compressed file size and Mean pixel value; B. Correlation between JPEG-compressed file size and Mean pixel value.

Our measures of visual complexity and luminosity were informed by recent research. Yet, other options are available [e.g., 39,40], and our choice should not be interpreted as a commitment to a specific method. We refer to the literature on the advantages and limitations of these approaches [see 39, about JPEG-compression]. However, please note both our measures of picture complexity show a robust negative correlation with luminosity (zip-compressed file size: r = −0.873, 95%CI [−0.906, −0.828], *p* < .00001, JPEG-compressed file size: r = −0.899, 95%CI [−0.926, −0.863], *p* < .00001; see Fig 1), suggesting that higher picture complexity corresponds to lower luminosity (i.e., more blackness). Therefore, our measures of visual complexity may broadly capture properties similar to those of the luminosity measure.

### Ratings

Ratings were collected in two separate studies, Study 1 and Study 2. Study 1 consisted of three tasks involving all the words and the corresponding pictures. Task ‘Pictures A’ focused on the pictures and had the goal of obtaining measures pertaining to: (i) Naming Consistency, i.e., how consistently a picture was named with a given label; (ii) Depictive Accuracy, i.e., to what extent the picture is a good illustration of the corresponding concept; and (iii) Familiarity ratings, i.e., how familiar participants are with the object presented in the picture. Task ‘Pictures B’ presented all the pictures again and was designed to collect ratings for the following dimensions: (i) Action Relatedness, defined as whether a concept is associated with an action that one can perform with their own body; (ii) Arousal, i.e., the degree to which the concept is arousing or exciting; and (iii) Valence, i.e., whether the concept is emotionally negative or positive. Finally, with the task ‘Words’ we collected ratings for the word items on (i) Action Relatedness, (ii) Arousal, and (iii) Valence. In all tasks, participants provided their ratings using a 1–7 Likert scale. Ratings 1 and 7 indicate, respectively, the lowest and highest levels of depictive accuracy, familiarity, action relatedness, and arousal; in the case of valence, 1 indicates negative valence, whereas 7 positive valence.

In Study 2 (‘Imageability’), we focused on the imageability of the word items. Given that all items of the database are concrete words for which high levels of imageability can be expected, we opted for a Visual Analogue Scale (VAS), providing a range of scores from 0 to 100, so that we could potentially better capture fine-graded differences between imageability of concrete concepts (compared to a 1–7 Likert scale).

### Participants

For both Study 1 and 2, inclusion criteria specified that participants had to be native speakers of German and be between 18 and 40 years old. Study 1 (Pictures A, Pictures B, Words) involved 39 participants (21 women, 18 men), with a mean age of 27.0 (SD = 5.4, range 20–38). Participants were distributed evenly across the three tasks. Study 2 (Imageability) involved 46 participants (25 women, 21 men) with mean age of 25.0 (SD = 4.4, range 18–35). All participants lived in Germany at the time of testing. There was no overlap between the participants of Study 1 and 2. They all signed written informed consent prior to taking part in the study and were paid for their participation. Data collection started on August 19^th^, 2022 and ended on March 20^th^, 2024.

### Procedure

In Study 1, participants received a link to participate in the study remotely, after being instructed about the procedures of the study. The tasks were administered and run using the online platform Penn Controller for IBEX [PCIbex, 44], which recorded participants’ responses. Participants were randomly assigned to one of the three tasks. At the beginning of the task, they were asked to sit in a quiet place and avoid distractions. They then received specific instructions about the task. For all three tasks, the pictures or words were presented one by one, in a random order. In task ‘Pictures A’, participants saw three subsequent events for each randomly presented picture. In the first event, they were asked to name the picture by typing the corresponding word. This was then followed by two events in which they provided, respectively, a depictive accuracy and a familiarity rating. In task ‘Pictures B’, participants saw three events, in which they were asked to rate each picture for, respectively, action relatedness, arousal, and valence. The ‘Words’ task was identical to ‘Pictures B’, except that words were presented instead of pictures. In all three tasks, the picture or word was on screen for all types of naming or rating events. Participants could not proceed to the next event or trial unless an answer had been provided.

Study 2 took place in a laboratory room of the Brain Language Laboratory at the Freie Universität Berlin. Participants saw each word with a vertical bar, the bottom and top ends of which represented the lowest (i.e., 0) and the highest (i.e., 100) imageability levels. They were instructed to move their cursor to the point of the vertical bar corresponding to the imageability level of each item and to click to give their assessment. After clicking, they would see the next item. The experiment was built and administered using the E-Prime 3.0 software [45].

The specific instructions participants saw in the tasks of both studies are provided at the project’s OSF directory (https://osf.io/k64nh/). The study was performed in accordance with the 1964 Declaration of Helsinki. The Ethics Committee of Charité Universitätsmedizin, Campus Benjamin Franklin, Berlin, Germany, approved all procedures (application Nr. EA4/102/21).

### Naming consistency

The database presents naming consistency for each item both as absolute values and as proportions out of the total number of responses. ‘Naming consistency’ here refers to the number of accurate responses, i.e., the number of responses consistent with the corresponding word of the database. This means that the naming consistency score might be low for some pictures, although participants named the pictures relatively consistently with a name that differed from the one we expected. This is because the purpose of the database is to have a set of pictures corresponding to a specific set of words, complying with the constraints regarding the initial phonemes described above under ‘Phonetic/phonological properties’. Responses that were almost equal to the target words except for misspellings or typos and plural forms of the target words were considered correct. We additionally scored the answer as accurate if it met both these two conditions: (i) the produced word had the same meaning as the target word (as determined by the third author, who is a native German speaker, and verified with a German dictionary) and (ii) the produced word was a more complex word fully containing the target word word-initially (e.g., *Panda-Bär* ‘panda bear’ instead of *Panda* ‘panda’), or both the produced and the target words were compounds and shared the same initial constituent. This is because the word could be considered equivalent to the expected word both semantically and phonologically (at least for the initial constituent) and was, therefore, compatible with our constraints. The database is accompanied by the raw naming data. Researchers needing to use our stimuli for other purposes may refer to those raw data and consider renaming the (few) pictures for which our naming consistency measure is low.

The naming consistency of the pictures in our database is generally high (1st quartile = 62%; median = 85%; 3rd quartile 92%; max = 100%). Two items (*Sardelle* ‘anchovy’ and *Schakal* ‘jackal’) have 0 naming consistency; we kept them in the database as they might be relevant for researchers using our stimuli for other purposes. Figure A4 in the Appendix presents the distribution of naming consistency values.

### Ratings results: general overview

For each item, we computed a mean value from the ratings of all participants for each of the rated dimensions (depictive accuracy, familiarity, action relatedness, arousal, and valence for pictures; action relatedness, arousal, valence, and imageability for words), as well as the SD of participants’ responses. Note that while we are aware of the limitations of treating rating scales as numerical values, and therefore computing a mean value [46], this is still the most economical approach (if not the only feasible) when establishing a database with norms obtained from a rating study, to be used to create matched experimental item sets or to be included as predictors in regression models. Indeed, similarly collected norms have been proved to successfully predict both behavior and brain imaging data [for some recent evidence, see 47–49]. Furthermore, the reliability and external validity results reported below provide additional indication that these mean values computed from the ratings are robust enough despite their limitations.

Below, we present a series of analyses of the mean ratings of the different dimensions. The goal is to provide a general overview and a validation of the database, which is intended as a resource to build sets of stimuli based on each researcher’s specific criteria. For this reason, for these analyses we did not distinguish between our pre-defined semantic and phonological categories, unless there was a theoretical motivation for doing so (see the comparison between animals and tools on action relatedness). Instead, mean ratings for the different semantic and phonological categories are presented in the section ‘Matched Stimulus Set’, which specifically focuses on building a stimulus set with different conditions starting from our database.

**Fig 2:**
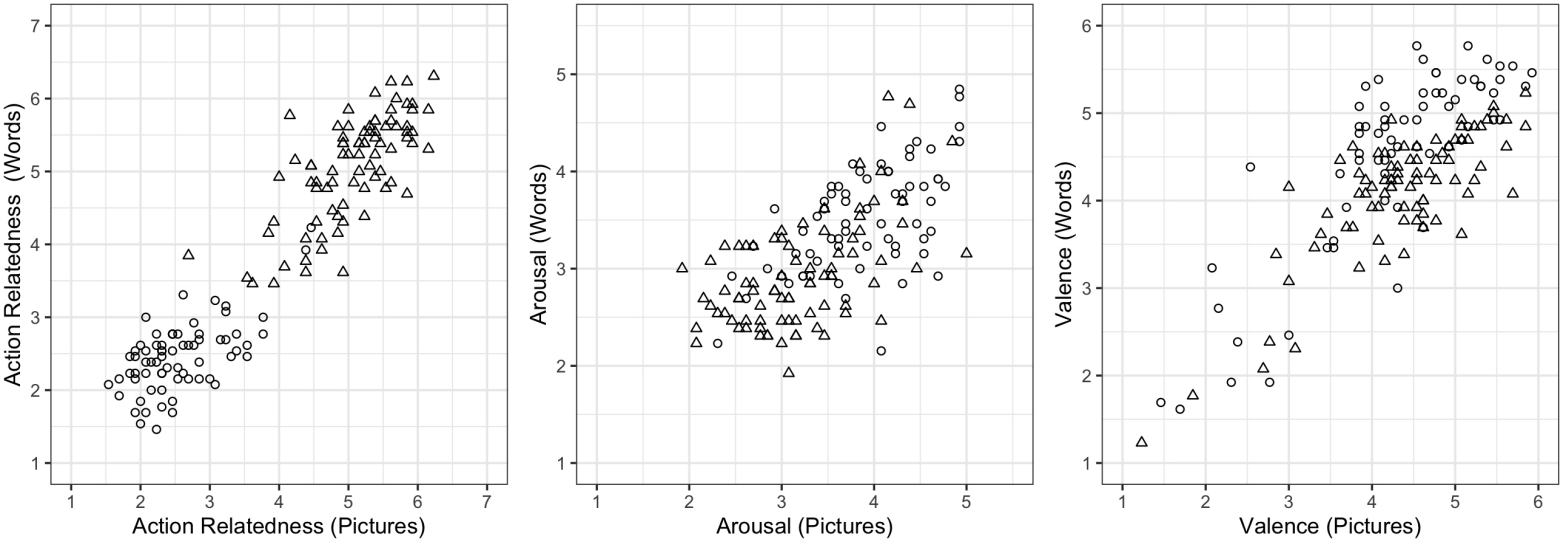
Correlations between action relatedness, arousal, and valence of the pictures and the corresponding words. Animal and tool items are visualized with different shapes (circles = animals; triangles = tools), to illustrate the different distribution of mean action relatedness values for the two semantic categories.

**Fig 3:**
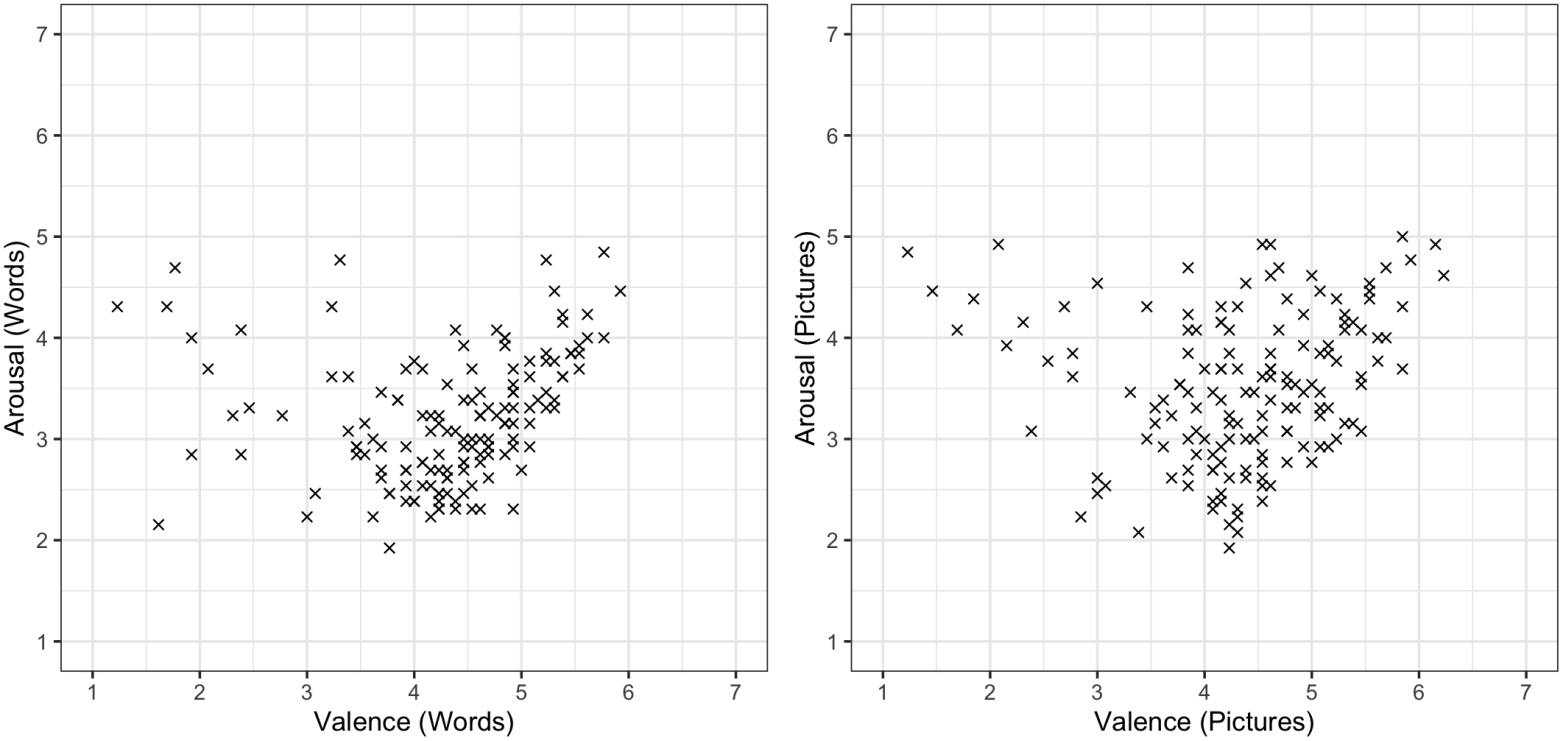
Relationship between pictures’ and words’ arousal and valence ratings. The x and y axes represent, respectively, valence and arousal mean ratings for each item; higher valence ratings stand for positive valence, while lower valence ratings represent negative valence.

Overall, depictive accuracy ratings of the pictures are high, with a mean of 5.92 (SD = 0.68; range: 3.23–6.92). Pictures’ familiarity ratings are slightly lower, with a mean of 5.48 (SD = 0.84; range: 2.23–6.92). Mean action relatedness is 3.92 (SD = 1.40, range: 1.54–6.23) for the pictures, and 3.87 (SD = 1.44, range = 1.46–6.31) for the words. Mean arousal and valence are, respectively, 3.51 (SD = 0.74, range 1.92–5.00) and 4.34 (SD = 0.94, range = 1.23–6.23) for the pictures, and 3.20 (SD = 0.63, range = 1.92–4.85) and 4.31 (SD = 0.91, range = 1.23–5.92) for the words. Word imageability is, as expected, very high, with a mean of 90.74 and low variability, considering that the potential range spans from 1 to 100 (SD = 7.96, range = 58.02–98.59). Figures A5 and A6 in the Appendix illustrate the distribution of pictures’ depictive accuracy and familiarity ratings and word imageability ratings, respectively. Action-relatedness, arousal, and valence ratings are visualized below as part of additional analyses (see Figs 2 and 3).

### Reliability

As initial sanity check for the quality of the ratings, we verified that tools received higher action-relatedness ratings than animals, as expected given their inherent association with actions. This was indeed the case both for the pictures (tools: mean = 5.06, SD = 0.67, range = 2.69–6.23; animals: mean = 2.56, SD = 0.61, range: 1.54–4.46), as also confirmed by a two-tailed t-test (*t* (149.51) = −23.716, *p* < .000001), and for the words (tools: mean = 5.06, SD = 0.72, range = 3.46–6.31; animals: mean = 2.46, SD = 0.49, range = 1.46–4.23), as confirmed by a two-tailed t-test (*t* (142.9) = −26.243, *p* < .000001). As additional verification of the quality of the data, we performed correlations between pictures’ and corresponding words’ values, for those dimensions for which we collected ratings on both picture and corresponding word (i.e., action-relatedness, arousal, and valence). We observed a positive correlation between each picture’s mean rating and that of the corresponding word (action-relatedness: r = 0.936, 95%CI [0.913, 0.953], *p* < .0001; arousal: r = 0.671, 95%CI [0.573, 0.750], *p* < .0001; valence: r = .784, 95%CI [0.714, 0.839], *p* < .0001), as illustrated in Fig 2. This confirms that our ratings captured the semantic similarity of these two classes of stimuli.

Furthermore, for both pictures and words, we observe a u-shaped relationship between valence and arousal (see Fig 3), as previously reported in several other studies [17,50,51; but see 52]. Following Sulpizio et al. [51], to confirm this relationship, we fitted a linear model predicting arousal from a linear effect of valence; the fit of this model was then compared, using the ‘anova’ function in R, to that of a model additionally including a quadratic effect of valence. For both pictures and words, the model that additionally includes the quadratic predictor provides a significantly better fit to the data (*p* < .0001 for both), therefore suggesting that a quadratic (u-shaped) relationship better describes the data than a linear relationship.

We finally checked for reliability of the data by means of a split-half reliability analysis, as a measure of internal validity, as done in previous similar studies [e.g., 53–55]. To this end, we divided the participant sample into two random halves and computed the mean rating for each word and dimension, separately for each of the two random halves. We then computed the split-half correlations between the scores of the random halves, corrected with the Spearman-Brown formula. We repeated this procedure 1000 times, each time generating different random halves from the sample, both for the ratings of Study 1 (picture depictive accuracy, picture familiarity, action relatedness, arousal, valence of pictures and words), involving 39 participants, and those of Study 2 (word imageability), involving 46 participants. Table 2 shows a summary of the results of this analysis. The results show strong correlations for all dimensions (all mean *r*s > 0.805) except arousal, for which correlations are rather moderate (mean *r *= 0.646 for words and 0.679 for pictures).

**Table 2:** Results of split-half correlations analyses: mean Spearman-Brown corrected r values and 95%CI of the 1000 correlation tests between the ratings from two random halves of the sample.

Dimension	Mean	Lower 95%CI	Upper 95%CI
Picture depictive accuracy	0.805	0.803	0.807
Picture familiarity	0.841	0.838	0.843
Picture action relatedness	0.932	0.930	0.934
Picture arousal	0.679	0.672	0.686
Picture valence	0.876	0.875	0.878
Word action relatedness	0.926	0.924	0.928
Word arousal	0.646	0.640	0.652
Word valence	0.873	0.872	0.874
Word imageability	0.951	0.951	0.952

### External validity

As measures of external validity, we compared our database to previously published databases of pictures or words in German, by computing correlations between our ratings and the ratings from the external resource (only considering those items that are contained in both). Regarding picture databases, the LinguaPix database [12] presents familiarity, arousal, and valence ratings. The two other available picture databases [11,13] do not include ratings for any of the dimensions we tested, and we could not find any German database featuring depictive accuracy, familiarity, or action relatedness ratings. The LinguaPix database includes 89 of our items. However, for some concepts, it contains more than one picture (from two to five), each rated separately. Consequently, there are a total of 123 items available for correlational analyses. For all three dimensions, we found significant positive correlations, though these are stronger for arousal and valence (respectively: r = 0.548, 95%CI [0.410, 0.661], *p* < .000001; r = 0.620, 95%CI [0.498, 0.718], *p* < .000001) than for familiarity (r = 0.226, 95%CI [0.051, 0.388], *p* = .012).

Concerning the word items, we checked for external validity of our arousal, valence, and imageability ratings with three different sources: the Berlin Affective Word List Reloaded (BAWL–R) [17], the ANGST, or German adaptation of the Affective Norms for English Words (German ANEW) [16], and the norms by Grandy et al. [15] (hereafter, GRANDY). The BAWL–R contains norms for arousal, valence, and imageability and includes 75 of our items. The ANGST/German ANEW contains ratings for all three dimensions too, with two types of ratings for arousal: those collected with the same method and scale as the BAWL-R, and those collected following the English ANEW. The database overlaps with ours in only 23 items. Finally, the GRANDY norms present ratings for imageability and valence (referred to as ‘emotionality’) from both younger (range = 21–31 years) and older (range = 70–86 years) participants and includes 109 of our items. We used the ratings from the younger group, as this best matches the age of our participants. None of these databases include ratings for action relatedness.

Our arousal ratings and those from the BAWL-R are highly positively correlated (r = 0.672, 95%CI [0.535, 0.780], *p* < .00001). Similarly, there is a positive, highly significant correlation between our ratings and the two arousal measures presented in the ANGST/German ANEW database, i.e., those produced following the BAWL-R method and those produced following the ANEW method (respectively: r = 0.872, 95%CI [0.717, 0.944], *p* < .000001; r = 0.723, 95%CI [0.443, 0.875] *p* = .000096). Concerning valence, our ratings are highly positively correlated with the ratings from all three databases (BAWL-R: r = 0.863, 95%CI [0.791, 0.911], *p *< .000001; ANGST/German ANEW r = 0.908, 95%CI [0.793, 0.961], *p *< .000001; GRANDY: r = 0.863, 95%CI [0.805, 0.904], *p *< .000001). Finally, there is a significant positive correlation between our imageability ratings and those from BAWL-R (r = 0.361, 95%CI [0.146, 0.543], *p* = .001) and from GRANDY (r = 0.630, 95%CI [0.501, 0.731], *p* < .000001), but no significant correlation with the ratings from ANGST/German ANEW (r = 0.238, 95%CI [−0.193, 0.592], *p* = 0.274).

### Matched stimulus set

In this section, we present a matched stimulus sets of pictures and corresponding words, which we created with the items of this database. It consists of 100 items, varying orthogonally for a semantic and a phonological variable, each with two levels: 48 items belong to the semantic category ‘animal’, while 52 are ‘tool’ items; similarly, 50 items belong to the PoA ‘coronal’ and 50 to the PoA ‘labial’. The two variables are crossed, so that 24 items are ‘animal-coronal’, 24 are ‘animal-labial, 26 are ‘tool-coronal’, and 26 are ‘tool-labial’.

To maximize the contrast between coronal and labial items, we only included items whose PoA of the second consonant is consistent with the PoA of the initial consonant, (e.g., ‘Fliege’ was excluded because/l/ is coronal while/f/ is labial). To achieve best possible naming performance in the experiment, we then only included items from the database with a naming consistency above 50%. Mean naming consistency of the resulting stimulus set is 82% (SD = 12%, range 54%−100%).

A summary of the items’ properties is presented in Tables 3–5. Note that, following Sassenhagen and Alday [56], we report descriptive statistics of the items without inferential statistics tests, as using such tests to prove good matching of a stimulus set is conceptually flawed [we refer to 56 for more details]. As can be seen from the tables, items were kept as similar as possible – across the two semantic categories, the two phonological categories, as well across the four subgroups crossing the semantic and phonological factor – for a series of lexical properties (length in letters, syllables, and phonemes; word form, lemma, and bigram frequency; Table 3) and semantic/conceptual properties (picture familiarity, depictive accuracy, valence, and arousal; word arousal, valence, and imageability; Table 4), as well as for naming consistency. Across the semantic dimensions, animal and tool items (both words and pictures) differ for the action relatedness ratings, since tools have, as expected, higher mean ratings than animals; instead, action relatedness is nearly perfectly matched across the words and pictures of the two phonological categories. Items across the different conditions were also controlled for several additional phonological and morphological properties (voicing of the first consonant, complex consonant clusters, syllables with complex onsets or codas, morphological complexity, and compounding; Table 5).

**Table 3:** Summary of the lexical properties of the matched stimulus set (mean, SD, and range).

		Number of letters	Number of syllables	Number of phonemes	Word form frequency (zipf)	Lemma frequency (zipf)	Bigram Frequency (zipf)
**Semantic Distinction**							
Animal	Mean (SD)	6.25 (1.74)	1.90 (0.63)	5.29 (1.52)	3.12 (0.62)	3.38 (0.63)	8.32 (0.30)
(N = 48)	min-max	4-11	1-3	2-8	1.61-4.47	2.03-4.88	7.58-8.76
Tool	Mean (SD)	6.33 (1.64)	2.12 (0.55)	5.48 (1.53)	3.23 (0.85)	3.40 (0.85)	8.37 (0.23)
(N = 52)	min-max	3-10	1-3	3-9	0.91-5.10	0.91-5.10	7.83-8.77
**Phonological Distinction**							
Coronal	Mean (SD)	6.42 (1.79)	2.04 (0.60)	5.54 (1.59)	3.26 (0.67)	3.45 (0.66)	8.36 (0.27)
(N = 50)	min-max	3-10	1-3	3-9	1.87-5.10	2.03-5.10	7.59-8.77
Labial	Mean (SD)	6.16 (1.58)	1.98 (0.59)	5.24 (1.44)	3.10 (0.81)	3.33 (0.82)	8.34 (0.27)
(N = 50)	min-max	4-11	1-3	2-8	0.91-4.47	0.91-4.88	7.58-8.76
Semantic * Phonological Distinction							
Animal Coronal	Mean (SD)	6.58 (1.72)	1.96 (0.62)	5.63 (1.53)	3.10 (0.58)	3.36 (0.59)	8.36 (0.29)
(N = 24)	min-max	4-10	1-3	3-8	1.87-4.16	2.03-4.32	7.59-8.76
Animal Labial	Mean (SD)	5.92 (1.74)	1.83 (0.64)	4.96 (1.46)	3.14 (0.68)	3.41 (0.67)	8.28 (0.31)
(N = 24)	min-max	4-11	1-3	2-8	1.61-4.47	2.03-4.88	7.58-8.76
Tool Coronal	Mean (SD)	6.27 (1.87)	2.12 (0.59)	5.46 (1.68)	3.41 (0.73)	3.54 (0.72)	8.35 (0.25)
(N = 26)	min-max	3-10	1-3	3-9	2.09-5.10	2.12-5.10	7.83-8.77
Tool Labial	Mean (SD)	6.38 (1.42)	2.12 (0.52)	5.50 (1.39)	3.06 (0.93)	3.25 (0.95)	8.39 (0.22)
(N = 26)	min-max	4-9	1-3	3-8	0.91-4.40	0.91-4.45	7.9-8.69

**Table 4:** Summary of the semantic/conceptual properties of the matched stimulus set (mean, SD, and range).

		Naming consistency (%)	Picture familiarity	Picture depictive accuracy	Picture arousal	Word arousal	Picture valence	Word valence	Picture action relatedness	Word action relatedness	Word imageability
**Semantic Distinction**											
Animal	Mean (SD)	86 (13)	5.28 (0.55)	5.94 (0.45)	4.05 (0.61)	3.64 (0.52)	4.46 (1.03)	4.73 (0.94)	2.65 (0.63)	2.54 (0.52)	92.69 (3.53)
(N = 48)	min-max	62-100	4.15-6.54	4.92-6.92	2.46-4.92	2.69-4.85	1.46-6.15	1.69-5.92	1.54-4.46	1.46-4.23	82.63-96.52
Tool	Mean (SD)	89 (11)	5.89 (0.75)	6.28 (0.48)	3.30 (0.71)	2.98 (0.55)	4.43 (0.94)	4.15 (0.77)	5.12 (0.69)	5.16 (0.65)	94.61 (3.37)
(N = 52)	min-max	54-100	2.62-6.92	4.92-6.92	1.92-5.00	1.92-4.69	1.23-5.85	1.23-5.23	2.69-6.23	3.46-6.31	76.50-98.59
**Phonological Distinction**											
Coronal	Mean (SD)	87 (13)	5.68 (0.76)	6.15 (0.52)	3.47 (0.78)	3.19 (0.68)	4.41 (0.76)	4.51 (0.71)	3.94 (1.46)	3.90 (1.45)	93.89 (3.62)
(N = 50)	min-max	54-100	4.15-6.92	4.92-6.92	1.92-4.92	1.92-4.85	2.08-6.15	2.46-5.92	1.54-6.15	1.69-6.23	82.63-98.59
Labial	Mean (SD)	88 (11)	5.51 (0.69)	6.07 (0.47)	3.84 (0.69)	3.41 (0.55)	4.48 (1.16)	4.35 (1.05)	3.93 (1.37)	3.90 (1.45)	93.48 (3.53)
(N = 50)	min-max	54-100	2.62-6.77	4.92-6.77	2.23-5.00	2.31-4.69	1.23-5.92	1.23-5.77	1.85-6.23	1.46-6.31	76.50-97.80
Semantic * Phonological Distinction											
Animal Coronal	Mean (SD)	84 (15)	5.13 (0.58)	5.83 (0.51)	4.01 (0.68)	3.64 (0.61)	4.38 (0.94)	4.74 (0.87)	2.53 (0.54)	2.49 (0.4)	92.14 (4.19)
(N = 24)	min-max	62-100	4.15-6.54	4.92-6.92	2.62-4.92	2.69-4.85	2.08-6.15	2.46-5.92	1.54-3.54	1.69-3.23	82.63-96.43
Animal Labial	Mean (SD)	88(11)	5.43 (0.48)	6.04 (0.36)	4.08 (0.54)	3.64 (0.42)	4.53 (1.12)	4.73 (1.01)	2.77 (0.70)	2.58 (0.62)	93.23 (2.71)
(N = 24)	min-max	62-100	4.54-6.54	5.23-6.54	2.46-4.77	2.92-4.31	1.46-5.92	1.69-5.77	1.85-4.46	1.46-4.23	86.57-96.52
Tool Coronal	Mean (SD)	90(11)	6.18 (0.53)	6.45 (0.32)	2.98 (0.49)	2.77 (0.43)	4.44 (0.56)	4.30 (0.45)	5.24 (0.47)	5.20 (0.56)	95.51 (1.99)
(N = 26)	min-max	54-100	4.77-6.92	5.77-6.92	1.92-3.77	1.92-3.62	3.31-5.46	3.38-5.08	3.85-6.15	4.15-6.23	89.52-98.59
Tool Labial	Mean (SD)	87(11)	5.59 (0.83)	6.11 (0.55)	3.62 (0.75)	3.20 (0.58)	4.43 (1.22)	4.01 (0.99)	5.00 (0.85)	5.12 (0.74)	93.71 (4.19)
(N = 26)	min-max	54-100	2.62-6.77	4.92-6.77	2.23-5.00	2.31-4.69	1.23-5.85	1.23-5.23	2.69-6.23	3.46-6.31	76.5-97.80

**Table 5:** Summary of phonological and morphological properties of the matched stimulus set (counts).

	Starts with voiced consonant	Starts with voiceless consonant	Starts with complex consonant cluster	Contains one or more syllables with complex onsets	Contains one or more syllables with complex codas	Is morpho-logically complex	Is a compound word
**Semantic Distinction**							
Animal (N = 48)	21	27	13	16	13	10	10
Tool (N = 52)	25	27	11	15	4	14	8
**Phonological Distinction**							
Coronal (N = 50)	23	27	17	19	10	13	9
Labial (N = 50)	23	27	7	12	7	11	9
Semantic * Phonological Distinction							
Animal Coronal (N = 24)	10	14	10	12	8	6	6
Animal Labial (N = 24)	11	13	3	4	5	4	4
Tool Coronal (N = 26)	13	13	7	7	2	7	3
Tool Labial (N = 26)	12	14	4	8	2	7	5

The stimulus set was used in an EEG study by Grisoni et al. [34] and is ready-to-use for any other researchers aiming to focus on the processing of semantic and phonological information and their interaction in language comprehension and/or production. It is available at the project’s osf repository: https://osf.io/k64nh/.

## Discussion and conclusions

In the present paper, we presented a database of 151 pictures and their corresponding German words. We provided an overview of the information included in the database, together with a summary of the main properties of the stimuli, both words and pictures. We then presented a series of analyses that we performed on the rating data that we collected. Particularly, we showed that our rating data: (i) are able to capture the semantic similarity between a word and the picture referring to the same concept, as indicated by the significant positive correlations between the words’ and pictures’ ratings; (ii) mirror previous reports of a u-shaped relationship between arousal and valence [17,50,51; but see 52], for both words and pictures; (iii) have good internal reliability, as suggested by the split-half correlational analysis; and (iv) have good external validity, indicated by significant correlations with the ratings from other existing resources for German [12,15–17].

Concerning the split-half correlational analysis, while the results overall suggest high internal consistency of the ratings, and therefore good internal reliability, the correlations are weaker for arousal than for the other dimensions, for both words and pictures. Note that weaker split-half correlations for arousal than other dimensions have been previously reported in similar studies [e.g., 54,55,57] and that, more generally, arousal ratings tend to be more variable across participants and populations [54,57]. This possibly reflects larger individual differences in perceiving this dimension, which is by nature more sensitive to life events or anxiety traits [see 54]. However, considering that the other analyses involving arousal show strong correlations in the expected direction (i.e., correlation between ratings for words and pictures; correlation with arousal ratings from other available resources), we can still conclude that all our ratings display good reliability and generalizability.

Coming to the comparisons with other existing resources, the overall results again suggest good external validity of our data, although some results warrant further explanation. First, we failed to find a significant correlation between our word imageability ratings and the ANGST/German ANEW database, while there is a significant correlation with the two other resources (GRANDY and BAWL-R). That the strongest correlation for imageability was found with the GRANDY database may be due to the similar scale (1–100) used in both studies, which allows for finer distinctions, especially for the semantic categories we included (animals and tools), which are both concrete and therefore highly imageable. Instead, besides the difference in scale, the lack of correlation with ANGST/German ANEW could at least partially be explained by the low overlap between the two datasets (23 items), which substantially reduces power. Regarding pictures, the familiarity ratings only weakly (but significantly) correlate with the LinguaPix database, while the correlations for the other dimensions with the same database are higher. A relevant difference between our database and LinguaPix is that the latter consists of color photographs, while we employed black-and-white line drawings. This difference in stimulus property might explain this result, as it might interfere more with perceived familiarity than with arousal or valence associated to a concept.

Finally, we presented a stimulus set obtained from our database, crossing a semantic (animal vs. tool words) and a phonological variable (coronal vs. labial), suitable for imaging studies addressing questions targeting differences in brain activations reflecting semantic and phonological distinctions, and, importantly, their interaction. We showed that the items of the different conditions are well balanced for a series of other lexical, phonological, morphological, and semantic/conceptual features. A quality criterion for such a stimulus set is that it should maximize the phonological contrast between target words of different phonological categories, by keeping all other factors equal. Therefore, ideally, target words should be phonologically identical, except for the initial, critical phoneme, i.e., they should be minimal pairs, such as *Wal* (‘whale’; labial) and *Tal* (‘valley’; coronal). Finding such minimal pairs in German is, however, challenging if not nearly impossible, especially considering that, for a picture naming task, it is important to select concepts that are easily picturable. Furthermore, to closely compare comprehension and production, the two words should ideally not only share all phonemes, but also all *graphemes*, except the first. When additionally considering the semantic constraint (animal vs. tool words), having minimal pairs was clearly not feasible. However, future efforts may look at whether this is at least possible for other languages, or for studies having fewer constraints (e.g., investigating auditory instead of written word comprehension).

Of course, the database only represents a first step towards creating larger resources and it comes with some limitations. Its main limitation is the relatively small number of items included, particularly in comparison with other available resources [e.g., 11,12,14]. This limitation is at least partly offset by the richness of information provided. Indeed, our database provides ratings and additional measures on a much larger set of variables than other resources. Importantly, to the best of our knowledge, this is also the first database including ratings on *both* pictures and corresponding words, across a series of relevant dimensions—an essential feature for studies focusing on both language production and comprehension. Finally, although other databases comprise larger numbers of items, they also tend to be quite heterogeneous, so that they do not necessarily include sufficient numbers of items for comparisons between specific semantic or phonological categories. Indeed, it was the fact that available resources for German do not include enough items for the categories we had pre-selected—as signaled by the relatively low overlap between our database and other available resources—that motivated the creation of this ad-hoc resource. Consequently, this database crucially complements existing resources by providing a tool that targets specific semantic (animals, tools) and phonological categories (based on PoA, MoA, or voicing), which was previously unavailable.

The decision to focus on stimuli referring to tool and animal concepts was based on available neuroimaging and neurophysiological findings, which suggest distinct neural responses for these two semantic categories. A possible next step would be to include items from additional categories, to allow for a larger number of possible comparisons. An example would be concepts that are largely experienced with senses different from vision, such as taste or smell (e.g., foods). These would be easily picturable and therefore easy to implement. Recent research has suggested that there might be a sensory hierarchy in human experience, with a predominance of vision and audition over other senses, which would have implications for how concepts are grounded in sensory experience [58–60]. Therefore, exploring concepts referring to different sensory modalities could be a potentially relevant direction. Furthermore, ideally, databases for word production research should also try to target abstract meaning; this will be the next challenge for picture naming databases, as even the largest currently available databases include only concrete items [11,12]. While recent studies using words have provided relevant resources to characterize abstract concepts [see, e.g., 61,62], representing them in pictures is less straightforward. It might not be feasible for some abstract categories, but starting with representations of social or communicative events might be the next step, particularly since these are two semantic categories that have been attracting growing interest in language research [e.g., 48,49,63].

Another relevant extension of the database would involve targeting additional languages, in a similar vein to the database by Duñabeitia et al. [11], to additionally allow for direct cross-linguistic comparisons of the dynamics involved in language production and comprehension and the time-course of semantics and phonology. For example, some recent studies have been targeting semantic and phonological distinctions in language production and comprehension in French [1]. Ideally, having a shared resource targeting the same concepts across multiple languages would allow for more comparability between different studies.

To conclude, the database represents a valuable tool allowing for well-controlled investigations of semantics and phonological processing and, importantly, their interaction, in both word production and comprehension. It adds to the relatively scarce list of available resources for German and can potentially be used or adapted for research on both healthy and language-impaired populations. Researchers interested in extending the database to more words or more semantic categories can find all relevant information, including the exact instructions we used for the rating studies, at our project OSF repository: https://osf.io/k64nh/.

## Supporting information

S1 Appendix Bar plots of the words’ length in syllables, letters, and phonemes. (TIF)

S2 Appendix Histograms of the words’ lemma, word-form, and bigram frequency. (TIF)

S3 Appendix Histograms of the pictures’ luminosity scores (mean pixel values) and visual complexity scores (file size of the zip compressed and JPEG compressed image). (TIF)

S4 Appendix Bar plot of naming accuracy scores (number of accurate picture naming responses). (TIF)

S5 Appendix Histograms of pictures’ depictive accuracy and familiarity ratings. (TIF)

S6 Appendix Histogram of words’ imageability ratings. (TIF)
